# The Effect of Sustainable Feeding Systems, Combining Total Mixed Rations and Pasture, on Milk Fatty Acid Composition and Antioxidant Capacity in Jersey Dairy Cows

**DOI:** 10.3390/ani12070908

**Published:** 2022-04-01

**Authors:** Anita Șanta, Daniel Mierlita, Stelian Dărăban, Claudia Terezia Socol, Simona Ioana Vicas, Mihai Șuteu, Cristina Maria Maerescu, Alina Stefania Stanciu, Ioan Mircea Pop

**Affiliations:** 1 Doctoral School of Agricultural Engineering Sciences, University of Agricultural Sciences and Veterinary Medicine, 3-5 Mănăștur St., 400372 Cluj-Napoca, Romania; anita.anita1403@yahoo.com; 2 Department of Animal Science, Faculty of Environmental Protection, University of Oradea, 1 University St., 410087 Oradea, Romania; clausocol@yahoo.com (C.T.S.); svicas@uoradea.ro (S.I.V.); cristina_maerescu@yahoo.com (C.M.M.); as1stanciu@yahoo.com (A.S.S.); 3 Department of Technological Science, Faculty of Animal Science and Biotechnologies, University of Agricultural Sciences and Veterinary Medicine, 3-5 Mănăștur St., 400372 Cluj-Napoca, Romania; ovineusamv@yahoo.com (S.D.); suteu_usamv@yahoo.com (M.Ș.); 4 Department of Animal Nutrition, Faculty of Animal Science, University of Agricultural Sciences and Veterinary Medicine of Iași, 3 Mihail Sadoveanu St., 700490 Iași, Romania; popmirceais@yahoo.com

**Keywords:** milk fatty acids, partial mixed ration, health lipid indices, antioxidants

## Abstract

**Simple Summary:**

Until now, only a limited number of studies have addressed the influence of combining grazing with total mixed rations (TMRs), in Jersey cows, on milk biocomponents and antioxidant capacity. Thus, the main objective of this study was to compare fatty acid (FA) profiles and antioxidant capacity in milk yielded by cows fed either using a TMR or a partial mixed ration (pTMR) combining a TMR and grazing. The second objective consisted in evaluating FA profiles and changes in lipophilic antioxidants in milk during the grazing period and related to pasture chemical composition. Although the pTMR feeding system led to a decreased daily milk production, milk quality was improved. By comparison, cows having access to pasture (pTMR group) yielded a milk with a higher fat and protein content, but with lower saturated FAs (SFA) and a higher content of unsaturated FAs considered benefic for human health (vaccenic acid (VA), conjugated linoleic acid (CLA), and omega-3 FA (n-3 FA)). Similarly, lipophilic antioxidant (α-tocopherol, retinol, and β-carotene) content was higher in the pTMR group, resulting in a higher total antioxidant capacity in milk. The grazing period showed a significant influence on milk quality; in the milk from the pTMR group, the highest concentrations of benefic FAs (VA, CLA, and n-3 FA), the highest concentration of fat-soluble antioxidants, and the best antioxidant activity, respectively, were obtained in the first grazing period (May), in accordance with the pasture’s chemical composition.

**Abstract:**

This study was carried out to assess the effect of using pasture combined with total mixed ration (TMR) on milk production and composition, fatty acid (FA) profiles, fat-soluble antioxidant content, and total milk antioxidant capacity (TAC). In addition, the effect of milk pasteurization and storage at 2 °C for 4 days, lipophilic antioxidants and TAC were assessed. Two homogeneous groups of Jersey cows (*n* = 8) were constituted, which were randomly assigned to one of the two treatments: TMR (without access to pasture), or partial mixed diet (pTMR: grazing 8 h/day + TMR indoors). To establish FA profiles and lipophilic antioxidants’ changes in milk during the grazing period, in case of the pTMR group the experimental period was spilt in three grazing periods: P_1_ (May), P_2_ (June), and P_3_ (June/July). Milk yielded from cows having limited access on pasture (pTMR diet) showed an improved FA profile, with higher concentrations of FAs considered benefic for human health (vaccenic acid (VA), conjugated linoleic acid (CLA), omega-3 FA (n-3 FA)) (*p* < 0.01) and a lower concentration of FAs with hypercholesterolemiant potential (C12:0, C14:0, and C16:0) (*p* < 0.05), compared to that of the TMR diet. This change in FA profile was correlated with improved sanogenous lipid indices of milk fat (n-6/n-3 FA atherogenic index and thrombogenic index). Milk yielded during the P_1_ grazing period had higher concentrations of VA, CLA, and n-3 FA (*p* < 0.05) and lower concentrations of C14:0 and C16:0 (*p* < 0.01); it exhibited the best values for the main sanogenous fat lipid indices of fat. Moreover, pTMR milk showed a higher α-tocopherol, retinol, and β-carotene content (*p* < 0.05), positively correlated with TAC values in milk (P_1_ ˃ P_2_ ˃ P_3_). By comparison, cows fed using the TMR diet yielded a higher quantity of milk (*p* < 0.05), but a lower fat and protein content (*p* < 0.01), and also a higher saturated FAs and n-6 FA content (*p* < 0.05) together with a lower concentration of lipophilic antioxidants in milk. Thermal treatment showed no effect on α-tocopherol and retinol content in milk, but their concentrations decreased during the storage, at the same time a TAC decrease. The results of this study prove the positive effect of using pasture combined with TMR on FA profiles, milk antioxidant content, and antioxidant capacity, with beneficial effects on nutrition and health in humans.

## 1. Introduction

A major interest in modifying milk fatty acid (FA) composition, aiming to enhance the health status of consumers, was noticed in the last two decades. Such changes are intended to decrease the proportion of saturated FAs (SFAs) in milk fat composition, and to increase the proportion of healthy FAs, such as vaccenic acid (VA), conjugated linoleic acid (CLA), and omega-3 FAs (n-3 FAs: α-linolenic acid (ALA), eicosapentaenoic acid (EPA), docosapentaenoic acid (DPA)), which are showing health benefits in humans [1].

Lately, milk FA profiles became increasingly less favorable for human health due to the cattle feeding systems, that use total mixed rations (TMR) based on silage feeds and high amounts of concentrates, and to a lesser extent based on the use of pastures [2].

The use of pastures in cattle feeding stands to reduce the feeding costs and to improve milk nutritional quality, most of all it being considered an environmentally sustainable production system, compared to the intensive ones in the shelter. The milk and the dairy products resulting from grazing are more appreciated by the consumers, being rated as more natural, healthier, and safer for human health, based on the natural environmental conditions enabled, which allow cattle to express their normal behavior [3,4]. The indoor housing of animals and the use of TMR-based diets for feeding (mainly consisting of silage feeds and concentrate mixtures) provides lower comfort for the animals and is associated with various diseases (mastitis, lameness, reproductive disorders), but also with decreased values of some biocomponents in the milk’s composition, which are considered benefic for the consumer’s health.

Seasonal variation in the floristic composition of pastures influences milk quality and, subsequently, the quality of the resulting dairy products. Milk obtained from pastures between April and October had a higher fat content compared to the rest of the year. Moreover, higher percentages of unsaturated FAs (C18:3n-3, CLA *c*-9, *t*-11, and *t*-11 C18:1) and lower percentages of C14:0 and C16:0 were reported during this period. Additionally, the resulting cheese scored better in almost all sensory attributes: odor, aroma, taste, and texture descriptors [5].

Elgersma [6] and Barca et al. [7] reported that the use of pastures in dairy cattle feeding leads to an increase in mono-unsaturated FAs (MUFAs) and polyunsaturated FAs (PUFAs) in milk fat, especially VA, CLA, and n-3 FA, and a decrease in FA concentrations with hypercholesterolemiant effect (C12:0, C14:0, and C16:0), compared to cattle fed using TMRs. However, feeding dairy cattle exclusively on pastures limits dry matter intake (DMI), which lowers milk production compared to the TMRs [8]. Therefore, pasture-based rations require supplementation with significant amounts of concentrates or with TMRs [9].

Pasture supplementation with a TMR is known as a partial mixed ration (pTMR; [10]), since pasture is not physically a part of the TMR [11]. Recent studies indicate that the pasture rations supplemented with TMRs in cows could facilitate the achievement of adequate DMI values, resulting in increased milk production, milk fat, and protein content, even in comparison to pasture-based diets supplemented with concentrates [9,12,13]. The pTMRs, compared to the pasture-based rations supplemented with concentrates, besides improving milk production, show a favorable effect on ruminal pH and rumen fermentation processes, which lead to increased ratios of nitrogen and energy use from feed [11].

Higher contents of unsaturated FAs in milk also require higher antioxidant levels (i.e., α-tocopherol, retinol, β-carotene), to reduce their oxidation [14]. The oxidative processes could lead to the decrement of milk nutritional quality and to a rise in unwanted flavors, which abate its psycho-sensory characteristics.

α-tocopherol and carotenoid concentrations in feed influence their concentration in the milk produced [15]. Pastures are a natural source of antioxidants and PUFAs (mainly ALA, C18:3 n-3) for dairy cows, directly influencing the milk content in these bioactive compounds [6]. The milk yielded from pasture, compared to that yielded from TMRs used in feeding cattle, has a higher content in terms of unsaturated FAs with health benefits (VA, CLA, ALA) and antioxidants (β-carotene, lutein, retinol, tocopherols, phytol) [4].

Although previous studies assessed the impact of grazing on milk production and composition [16,17], there are still only a limited number of studies related to diets combining pasture with TMRs head-to-head with TMR feeding systems [11], especially in terms of lipophilic antioxidants and FA profiles of milk in relation to its TAC. Additionally, it is important to ascertain whether limited-time grazing can improve milk content in unsaturated FAs (mainly n-3 FA, VA, and CLA) and if the risk of their oxidation is negated by the higher concentrations of lipophilic antioxidants (mainly α-tocopherol, retinol, and β-carotene) available in pasture and further transferred into the milk. Therefore, the present study aimed to evaluate the impact of grazing 8 h a day and diet supplementation with a TMR, provided *ad libitum,* indoors (pTMR), in comparison to the indoor feeding system based on TMRs, on milk production and composition, biocomponents content, and TAC of milk in Jersey cows. In this study, the following hypotheses were assessed: (1) The expectancy of important changes in the chemical composition, FA profile, non-enzymatic lipophilic antioxidant content and TAC in raw milk and milk after pasteurization and storage; (2) The nutritional and sanogenic quality of milk yielded in the pTMR system (pasture + TMR) depends on the periodic variations in the chemical composition of the pasture.

## 2. Materials and Methods

All experimental procedures have been evaluated and approved by the Ethics Commission of the Faculty of Environmental Protection (3/25/01/2022) of the University of Oradea (Romania) in agreement to the European Union Directive number 2010/63/EU on the welfare and protection of animals used for scientific purposes.

### 2.1. Experimental Design and Treatments

The study was conducted at the University of Oradea (Romania) during 19 April and 11 July 2021. In total, 16 mid-lactation Jersey dairy cows were chosen. They had an initial average weight of 525 ± 49 kg (standard error of mean), 2.3 ± 1.1 lactations, 138 ± 57 days of lactation, and an average daily milk production of 25.7 ± 3.7 kg. The cows were divided into two homogeneous groups of eight cows and randomly assigned to two different feeding systems: TMR (TMR provided indoors, without grazing time), or pTMR (grazing + TMR provided indoors). Cows on pTMR diet were pasture-fed for 8 h/day between the morning and the evening milking time. The same TMR mixture was used in both treatments, being provided ad libitum, indoors. The TMR contained a mixture based on corn silage and concentrates (% of DM: corn silage—42.0; alfalfa hay—10.0; grass hay—7.3; corn grain—10.0; triticale—5.0; soybean meal—9.0; rapeseed meal—12.0; sodium bicarbonate—0.3; salt—0.4; vitamin and mineral premix—4.0), and it was balanced for net energy and protein as recommended by NRC [18].

The TMR composition used in cattle feeding, according to the two experimental groups, was formulated in agreement to NRC disclosures [18] for a milk production of 26 kg/day and a feed intake of 21.8 kg DM/head/day, considering a refusal rate of 10%. The TMR ensured a 60/40, forage/concentrate ratio.

All cows were milked twice a day: between 07:00–08:00 and 17:00–18:00, respectively. After the morning milking, cows from the pTMR group were moved on the pasture until the evening milking time, and cows from the TMR group were housed in shelter during the entire experimental period.

The experimental period ran for 84 days (12 weeks), of which the first week was considered as a pre-experimental period, when the experimental groups were constituted, followed by a two-week adaptation period of the cows on pasture and a period for recording the evidence of productive performances and sampling of 9 weeks. The experimental period, for the pTMR cattle group, was divided into three grazing periods (sampling periods—P) of 3 weeks each: P_1_ (May: 10–30 May), P_2_ (June: 31 May–20 June), and P_3_ (June/July: 21 June–11 July).

All animals, both housed in shelter and on pasture, had free access to fresh water.

### 2.2. Experimental Procedure

A pasture consisting of a land area of about 11.5 ha was split into 7 equal grazing land parcels, delimited using a mobile electric fence. In March, before starting the experiment, 60 kg of N/ha were applied, in form of urea, for pasture fertilization. The grazing time per one land parcel was of 3 days, using three rotational grazing time periods per parcel along the study. Thus, the cattle returned to the same pasture parcel after a three-week period of grass regeneration.

The floristic composition of the pasture was assessed, before grazing, by cutting the plants at ground level using scissors, from four different areas of 0.25 m^2^/each parcel. A green mass production from the pastures of about 2420 kg DM/ha was estimated based on the amount of grass sampled, which was afterwards weighed. Species identification was carried out using standard keys for plants identification. Thereby, the specific share of various plant species (number of individuals of each species related to the total number of determined individuals) from the pasture floristic composition was calculated. The pasture composition in the first grazing period (P_1_) consisted of about 22% *Lolium perenne*, 16% *Dactylis glomerata*, 10% *Festuca arundinacea*, 8% *Phleum pratense*, 8% *Poa pratensis*, 4% *Poa trivialis*, 24% *Trifolium repens*, and 4% weeds (*Taraxacum officinale*, *Plantago lanceolata*, *Veronica chamaedrys*, *Trisetum flavescens*). Dead matter accounted for about 4.0%. In the P_2_ and P_3_ grazing periods the white clover (*Trifolium repens*) percentage gradually decreased down to about 15% and the percentages of grasses (especially *Poa trivialis* and *Phleum pratense*), including weeds, increased.

On the first and the last day of each experimental period (e.g., after 21 days), after the morning milking, all cows were individually weighed. The cows were mechanically milked twice a day, and the daily milk production was recorded per individual by totalizing the production yielded at the two milking times.

The cows in the TMR group, received the TMR twice a day, after the morning and the evening milking, respectively, while the cows in the pTMR group received the TMR mixture after the evening milking. The unconsumed TMR mixture was considered refused and was removed from the stable once daily (07:00). The TMR intake was recorded daily in the two cattle groups, by gravimetric means. Estimated pasture DMI was determined as the amount of pasture necessary to supply the difference between the net energy (NE) requirements of the cows and that provided by TMR, using NRC prediction equations [19]. Energy requirements were recorded as NE (Mcal/day) requirements for maintenance, lactation, body weight (BW) changes, walking, and grazing. The average NE requirements of the cows were estimated using NRC [18] prediction equations:

- For maintenance: NE_L_ (Mcal/day) = 1.1 × (0.080 × BW^0.75^);
- For lactation: NE_L_ (Mcal/day) = milk production (kg/day) × (0.3512 + [0.0962 × %milk fat]);
- For body weight changes: daily weight gain = 5.12 Mcal/kg BW; while average daily weight loss = 4.92 Mcal/kg BW;
- For walking to the pasture and back to the shelter: 0.62 cal/(kg BW × m). Distance measurements to each parcel were performed and showed a mean value of 752 m (one way);
- For grazing: NE_L_ (Mcal/day) = 1.2 kcal × 8 h × BW^0.75^.

### 2.3. Sample Collection

Biomass was sampled weekly from the pasture, on the first day, before moving the cows to a new grazing parcel. Pasture sample were harvested from four different areas, by cutting, using scissors and a stubble was left of about 2–3 cm. All samples were amassed in a single sample for each grazing period. TMRs were sampled weekly during the experimental period and at the end, all samples were also amassed in a single sample for the laboratory assays. From these, 0.2 kg were sampled of each one, packed in plastic bags, and then kept at −20 °C until the subsequent laboratory tests were performed.

The milk samples were collected weekly, on the second day of grazing on a new parcel, making individual composite samples, consisting of the milk yielded at the two milking times (in the morning and in the evening) and taking into account the milk yielded at each milking ratio in achieving the total daily milk production. The milk samples were gathered in parallel from the cows of the two experimental groups. Two milk samples were collected from each individual: one quickly transported to the laboratory for chemical composition assays and one further frozen and kept at −20 °C until the assessment of FA profiles.

Two milk samples were collected from each cow in the experiment, in the 3rd, 6th, and 9th week; one of them was frozen and kept at −80 °C, and then analyzed for retinol (vitamin A), α-tocopherol (vitamin E), and β-carotene content [20], and the second one was quickly brought to the Laboratory of Chemical Analyses for TAC assessment. Milk pasteurization was carried out in glass tubes, in a water bath, at 65 °C for 30 min, samples were stirred up manually at five-minute intervals. Subsequently, the samples were cooled in cold water and then stored at 2 °C in a household refrigerator for 4 days. At the same time, fodder samples (TMR and pasture, 3 repetitions/sample) were collected along with the milk ones, being further frozen and stored at −20 °C until laboratory analyzes were carried out for α-tocopherol and β-carotene content assessment.

The samples were transported to the Laboratory of Chemical Analyses using refrigerated boxes, which prevented any alteration of quality.

### 2.4. Feed and Milk Chemical Analyses

#### 2.4.1. Feed and Milk Composition

The fodder (TMR and pasture) chemical composition was assessed in agreement with AOAC statements [21]. Dry matter content was assessed using the gravimetric method; crude protein content, using the Kjeldahl method (N × 6.25); crude fiber content, according to the manufacturer specifications (VELP FIWE 6, VELP Scientifica Srl, Usmate Velate (MB), Italy); ether extract content (SOXTHERM, C. Gerhardt GmbH, Königswinter, Germany) and total ash content, using the gravimetric method. The chemical analyzes were performed in the Laboratory of Animal Nutrition and Biochemistry of the University of Oradea (Romania).

The analysis of the chemical composition of milk was carried out using MilkoScan 7 RM and Fossomatic cell counter 7 (Foss Electric, CombiFoss 7, FOSS, Hilleroed, Denmark) by infrared spectroscopy (IDF Standard 141C, International Dairy Federation: Brussels, Belgium, 2000).

#### 2.4.2. Feed and Milk Fatty Acid Profiles

The FA composition was assessed both in milk and feed samples (TMR and pasture) and consisted in two stages: trans-esterification/methylation of FAs and separation and identification of methyl esters by gas chromatography [22]. The fat was extracted according to Folch et al. [23], and the FA methyl esters (FAME) were prepared by means of trans-methylation using methyl alcohol. The lipid extract was solubilized in a volume of 3 mL chloroform, next 3 mL of benzene, 3 mL of BF3, and 1.5 mL of methanol being further added. The mixture was warmed up at 80 °C for 2 h, afterwards 6 mL hexane and 3 mL of distilled water were added. After that, the sample was homogenized using a vortex.

The upper organic phase was collected and passed into to a test tube, containing anhydrous Na_2_SO_4_. Further, 1 mL hexane and 1 mL of water were added once again to the sample and then vortexed. Hexane extract evaporation was carried out using a rotary evaporator, 1.5 mL hexane being added to the resulting residue. Methyl esters’ separation in milk and feed samples (TMR and pasture) was made by means of Varian 3800 GC-4000as chromatograph (Varian, Inc. CA 94598-1675/USA) with a CP-Sil 88 column (100 m × 0.25 mm, 0.20 μm BC; Varian, Inc. CA 94598-1675/USA)). Helium was used as carrier gas to a flow rate of 1 mL/min. The injector and detector temperatures were set to 250 °C and 260 °C, respectively. The analysis operating parameters were: column initial temperature at 140 °C for 5 min; gradient temperature at 3 °C/min up to 200 °C and plateau at 200 °C for 5 min; gradient temperature at 3 °C/min up to 240 °C, plateau at 240 °C for 40 min.

The FA methyl esters’ identification from the analyzed samples (milk and feed) was performed by comparing their retention times to those of the pure methyl esters standard (Matreya Inc., State College, PA, USA; Sigma-Aldrich Inc., Burlington, MA, USA). At the outset, FA methyl esters standards were injected into the gas chromatograph to enable retention time identification for each one.

#### 2.4.3. Feed and Milk Fat-Soluble Antioxidants

For the α-tocopherol and β-carotene assessment in feed (TMR and pasture), 5 g of feed were weighed and mixed with the extraction solvent [24]. A mixture of hexane and ethanol (1:1, *v*/*v*) was used as a solvent. After filtration, saponification was carried out with a potassium methanol solution under nitrogen atmosphere. Subsequently, the two phases were separated in a separatory funnel by adding distilled water and diethyl ether. Distilled water was used to wash the organic phase, that was further dried in a stream of nitrogen gas. Next, 100 μL of ethyl acetate was added for the high-performance liquid chromatography (HPLC) assay; the method is similar to the one used for milk.

Retinol and α-tocopherol concentrations in milk were assessed by means of HPLC, after the pre-extraction of compounds by standard method [22]. Then, 500 μL of milk and 400 μL of pyrogallol were mixed in a tube and vortexed. Next, 1 mL of distilled water and 600 μL of ethanol were added and homogenized using a vortex. The extraction steps were repeated twice. Further, 4 mL of hexane were added, being then mixed and centrifuged (2000× *g* for 10 min). Then, 3 mL of the organic phase were sampled and evaporated under nitrogen. Antioxidants (β-carotene, retinol, and α-tocopherol) assay was performed by HPLC (Agilent Technology series 1100, Santa Clara, CA 95051, United States) using a Phenomenex Sphereclone column (5 μm, 150 × 4.6 mm) (Santa Clara, CA 95051 United States). The column was washed by adding MTBE:MeOH (50:50) after each assay performed. Methanol was used as mobile phase to a flow rate of 1.3 mL/min for 8 min. Chromatograms were monitored at 450 nm for β-carotene, 340 nm for retinol, and 290 nm for α-tocopherol. The compounds were identified using standards having a purity >90% (β-carotene: C4582; trans-retinol: R7632, and α-tocopherol: T3251) (Sigma-Aldrich, Madrid, Spain). Quantification was carried out using regression analysis of calibration curves of known standards. Duplicate analyses were performed for all samples.

#### 2.4.4. Milk Antioxidant Capacity Analysis

TAC was assessed using the ABTS method (2,20-azino-bis [3-ethylbenzothiazolin-6-sulfonic acid]) as described by Chen et al. [25], based on an adapted protocol, as described by Mierliță and Vicas [26]. In brief, ABTS^•+^ cation radical was produced by reacting ABTS solution 7 mM with potassium persulphate solution 2.45 mM, the mixture being kept in dark for 12 h. The resulting ABTS solution was diluted using a phosphate buffer, pH 6.7, until the absorbance at 730 nm was 0.70 ± 0.02. The calibration curve was achieved by using Trolox (water-soluble vitamin E analogue) as standard, at a concentration range of 1–500 μM (R^2^ = 0.9989). Milk samples were diluted 10 times. After the addition of 100 μL of sample to 2400 μL of ABTS cation radical diluted solution, the absorbance is read at 730 nm after precisely 1 min. A blank sample was used for each milk type to enable residual turbidity correction. The antioxidant capacity of milk was expressed as µmol Trolox equivalent (TE)/mL.

### 2.5. Nutritional Indices and FA Ratios

The ratios of n-6/n-3 FAs, hypocholesterolemients/Hypercholesterolemitants FA (h/H FA) ratio, the atherogenic index (AI), the thrombogenic index (TI) and the health promotion index (HPI) are commonly used to assess the nutritional quality of milk fats in terms of the impact on human health. To calculate the nutritional indices of milk fat, the following equations were used [22]:

- n-6/n-3 FA = (C18:2n-6 + C18:3n-6 + C20:4n-6)/(C18:3n-3 + C20:5n-3 + C22:3n-3 + C22:5n-3 + C22:6n-3);
- AI = (C12:0 + (C14:0 × 4) + C16:0)/(MUFA + PUFA);
- TI = (12:0 + 16:0 + 18:0)/[(0.5 × MUFA) + (0.5 × n-6FA) + (3 × n-3FA) + (n-3FA/n-6FA)];
- HPI = (n-3 PUFA + n-6 PUFA + MUFA)/[C12:0 + (4 × C14:0) + C16:0];
- h/H FA = (C18:1 + PUFA)/(C12:0 + C14:0 + C16:0).

∆9-desaturase is responsible for the enzymatic desaturation processes occurring in the mammary gland. This enzyme activity can be indirectly estimated based on the ratio: (product of ∆9-desaturase)/(product of ∆9-desaturase) + (substrates of ∆9-desaturase). The desaturase index (DI) was calculated according to Kay et al. [27]:

DI = (14:1 + 16:1 + *c*9,18:1 + *c*9,*t*11 CLA)/(14:0 + 14:1 + 16:0 + 16:1 + 18:0 + *c*9,18:1 + *t*11,18:1 + *c*9,*t*11 CLA).

### 2.6. Statistical Analysis

The experiment was designed and conducted as a randomized complete block with repeated-measures analysis over time. For statistical analysis, the data were analyzed using the SAS software [28]. The statistical significance of the results for milk production and composition, FA profiles, antioxidant content, and TAC of milk was assessed using ANOVA with repeated measures and factorial term for diet type. The statistical model included feed type (TMR, or pTMR) and grazing period (P_1_, P_2_, and P_3_) as fixed effects. The data collected indoors in shelter at the beginning of the study related to the chemical composition and production of milk were included as co-variables in the statistical model. The grazing periods were considered repeated factors, having cows as subjects. The results are presented as mean values and standard errors of the mean (SEM). Multiple comparisons between means were performed using Tukey’s test. The significant results were considered at *p* < 0.05 values.

## 3. Results

### 3.1. Chemical Composition of the Forages

The chemical composition, the FA content, and the fat-soluble antioxidants in the TMR and pasture, for each grazing period, are described in Table 1. The TMR, in comparison with the pasture grass, showed a higher content of DM, crude protein, and nitrogen free extractives (NFE), but a lower crude fiber. The chemical composition of the pastures changed during the experimental periods. During the first grazing period (P_1_), the grass biomass showed the lowest DM and crude fiber content, and the highest crude protein content (N × 6.25), while the highest levels of DM and crude fiber and the lowest crude protein content, respectively, were achieved in the last grazing period (P_3_) (Table 1). Therefore, the NE_L_ (Mcal/kg DM) was lower for the pasture than for the TMR, and on the pasture it decreased from P_1_ to P_3_. The fat content did not show significant differences related to the TMR and the pasture, nor to the three grazing periods.

The pasture showed a higher content of PUFAs, mainly of ALA (C18:3 n-3), while in the TMR a higher content of linoleic acid (LA, C18:2 n-6) and oleic acid (OA, C18:1 *cis*-9) was seen. The content for the majority of FAs (in particular C16:0 and C18:2 n-6) increased at the same time as plants’ growth stages (P_1_ ˂ P_2_ ˂ P_3_), except for ALA (C18:3 n-3), its concentration gradually decreased from 68.10 (% of total FAs) to 60.54% (*p* < 0.001). The ratio 18:2/18:3 was of 4.6:1 in the TMR and of 0.15–0.23:1 in pasture, being more favorable in the first grazing period (P_1_).

The mean values for α-tocopherol and β-carotene (all-*trans* β-carotenes) content in the TMR and in the pasture are also presented in Table 1. All-*trans* β-carotenes were the main antioxidants in the pasture, meanwhile the TMR showed a low content of antioxidants. In comparison to the TMR diet, the pasture content in carotenes was 10 times higher (6.73 vs. 66.97 mg/kg DM), and in α-tocopherol 3–4 times higher (8.64 vs. 20.1–34.4 mg/kg DM). The antioxidants level in grass was higher during the first grazing period (P_1_).

### 3.2. Dry Matter Intake, Milk Yield, and Milk Composition

The total DMI (kg DM/day) was not influenced by the cow’s feeding system (Table 2). The TMR intake in the pTMR group of cows, which had access to the pasture compared to the TMR group of cows, decreased significantly (18.11 vs. 13.80 kg DM/day) (*p* ˂ 0.001). Additionally, in the pTMR group of cows, the lowest TMR intake was achieved in the first grazing period (*p* ˂ 0.05), when the pasture intake (kg DM/cow) had the highest values across the entire experimental period (P_1_ ˃ P_2_ ˃ P_3_) (*p* ˂ 0.01).

The live body weight of cows shifted along the experimental period by +9 kg/head in the TMR group and by −7 kg/head in the pTMR group, showing significant differences.

Milk’s production and composition are presented in Table 3. The milk production was significantly lower in the pTMR cows, but it had a higher content of fat, urea, and somatic cells and a lower content of lactose, compared to that obtained from the cows in the TMR group. No significant differences were found between treatments in terms of corrected milk production (fat-corrected milk, energy-corrected milk) and total fat, protein, and lactose content, respectively. The mean milk production decreased during the grazing season, as expected (Table 3). Protein and lactose content in milk did not show significant changes during the three grazing periods, while fat content increased (4.78–5.21%), and the content of urea and somatic cells decreased (*p* ˂ 0.05).

### 3.3. Milk Fatty Acids Profile

FA composition of the milk fats, related to the feeding system and to the grazing period, is shown in Table 4.

The feeding system (TMR vs. pTMR) significantly altered the FA profiles in milk. The milk yielded by the cows fed using the pTMR diet had lower SFA concentrations (*p* < 0.01), higher MUFAs (*p* < 0.01), and PUFAs (*p* < 0.01) concentrations, compared to the milk produced by the cows fed exclusively indoors, with the TMR. The grazing used in the case of the cows from the pTMR group lead to a decrease of 11.0% in hypercholesterolemiant FA content (the sum of C12:0, C14:0, and C16:0) of milk fat and to an increase of 13.3% and 23.6%, respectively, in MUFA (mainly C16:1 *cis*-9, C18:1 *trans*-11, and C18:1 *cis*-9) and PUFA (mainly C18:2 n-6, CLA, and C18:3 n-3, respectively) content in comparison with the TMR diet (*p* < 0.01).

Out of the SFAs, the myristic acid (C14:0) and the palmitic acid (C16:0) showed higher levels in the TMR milk (*p* < 0.05), while the stearic acid (SA, C18:0) had a higher level (*p* < 0.05) in milk yielded by the pTMR group cows. Additionally, the *trans*-VA (C18:1 *t*-11) and the ALA (C18:3 n-3) levels were significantly higher (*p* < 0.001) in the milk yielded by the cows fed using the pTMR diet compared to that yielded by the animals fed indoors using the TMR diet, in which, milk showed rather a higher concentration of LA (C18:2 n-6) (*p* < 0.05). Thereby, C18:2 n-6 content in the TMR cows’ milk showed a value with 23.08% higher than that of the milk from pTMR cows. The CLA (C18:2 *c*-9, *t*-11) content showed two times higher values in the milk from cows fed on pasture (pTMR group) compared to that in the cows from the TMR group (*p* < 0.001).

The FAs exceeding 20 atoms are found in small amounts in milk fat. The pasture (pTMR group) enabled a significant increase in C20:5 n-3 (EPA) and C22:5 n-3 (DPA) content (*p* ˂ 0.05).

The cows that had access to the pasture (pTMR group) showed an enhanced quality of milk fat, in terms of human health impact, compared to the cows fed indoors using only the TMR. Thus, the PUFA/SFA ratio (0.11 vs. 0.08), h/H FA ratio (h/H: 0.85 vs. 0.65), and HPI significantly increased (*p* ˂ 0.05), whereas AI (*p* ˂ 0.05), TI (*p* ˂ 0.01), and n-6/n-3 FA ratio (*p* ˂ 0.01) decreased (Table 5).

In our experiment, the pTMR milk’s fat showed a 2.5 times lower n-6/n-3 FAs ratio than in the TMR milk, where the ratio was above 4.

The grazing period had a significant influence on the FA profile in milk. The highest PUFA level and the lowest SFA level were recorded in the first grazing period (P_1_) compared to the P_2_ and P_3_ ones. A 7.50% total content of PUFAs (% of total FAs) was recorded in the first grazing period (P_1_), C18:2 n-6 (2.90%), C18:2 *c*9, *t*11 (2.49%), and C18:3 n-3 (1.54%) being the most representative ones. The total PUFA values decreased in the second period showing almost the same values in the third grazing period. CLA (C18:2 *c*9, *t*11) and ALA (C18:3 n-3) showed a relatively similar trend, while the LA (C18:2 n-6) had a gradual increment, with a maximum level in the third period (3.44% of total FAs). The milk yielded during the P_1_ grazing period showed higher concentrations of *c*9, *t*11-CLA, C18:3 n-3, and lower concentrations of C14:0 and C16:0, than the ones registered in the P_2_ and P_3_ period, which had the best values for the main sanogenic lipid indices of fat: PUFA/SFA, n-6/n-3 FA, AI, TI, h/HFA, and HPI.

### 3.4. Fat-Soluble Vitamins and Antioxidant Capacity

Cows’ feeding regimen had a significant influence on the fat-soluble antioxidants mean content in milk. The milk fat of the cows grazing 8 h/day (pTMR group) showed α-tocopherol (0.74 g/100 g), retinol (125.62 μg/100 g), and β-carotene (0.69 μg/100 g) higher concentrations, compared to that in milk yielded by the cows fed indoors using the TMR (0.27 mg/100 g; 57.51 μg/100 g, and 0.41 μg/100 g, respectively). The content of lipophilic antioxidants in milk is linked with the TAC value. The TAC value was higher in the pTMR group both in the raw and the pasteurized milk, specifically the milk pasteurized and stored in the refrigerator for four days (3.02 µmol TE/mL, 2.73 µmol TE/mL, 2.58 µmol TE/mL, respectively) compared to the TMR group (2.53 µmol TE/mL, 2.47 µmol TE/mL, 2.40 µmol TE/mL, respectively) (Table 6). Thermal treatment showed no effect on α-tocopherol and retinol content in milk, but the storage of the pasteurized milk in the refrigerator for four days had a negative effect on these natural antioxidants.

The pasteurization had a negative effect on TAC, significant differences being pointed out only in the case of the pTMR group. The TAC of the pasteurized milk obtained from the two groups of cows did not change after the four days storage period. However, the TAC of the milk produced by the cows fed indoors with the TMR was lower compared to that of the pTMR milk, after the four days storage period, even if the differences were not statistically significant. The TAC of the pasteurized milk yielded by the two groups of cows did not show changes after a four days storage period. However, the TAC in the milk yielded by the cows fed indoors using the TMR was lower compared to that in the pTMR milk, after a storage period of four days, even if differences are not statistically significant.

## 4. Discussion

### 4.1. Chemical Composition of the Forages

The TMR’s chemical composition was linked to its composition. The TMR, consisting of corn silage (42.0% of DM), hay (17.3% of DM), and concentrates (40.7% of DM), had a content of 17.2% crude protein and 15.8% crude fiber. The OA (18:1 *c*9) and the LA (18:2 n-6) were the most representative FAs in the TMR fat composition, originating from the fat composition of the corn silage and grains [26].

The pasture biomass’ chemical composition has changed across the three grazing periods, as expected. The results of this study related to the chemical composition of biomass from pastures are in line with those reported by Radonjic et al., [29] and Bohacová et al. [30], who stated that the pasture content in crude fiber increased with the plants’ vegetative growth, while the protein content decreased. According to this study, a strong positive global linear relationship was ascertained between the crude protein and the ALA (C18:3 n-3) content in pasture [6]. The DM content increase in the pasture, is mainly due to the crude fiber increment, resulting in a reduction in energy content, which is the main limiting factor for the milk yielded from pasture [31].

The carotenoid and tocopherol content of the fodder (TMR and pasture) ascertained in our study is in agreement with that of Cabiddu et al. [32], who claim that it is influenced by the plant age (gradually decreasing from the plant vegetative to the reproductive stage), the preservation method (fresh ˃ silage ˃ hay) and the botanical composition of the pasture.

### 4.2. Feed Intake, Milk Yield, and Milk Composition

In the present study, no significant differences were noticed related to the DMI between the TMR and pTMR cows. However, milk production was significantly lower in the pTMR cows, probably due to the low energy intake of pasture and energy costs implied by the walk to pasture, and by grazing. Opposite to our results, studies conducted by De La Torre-Santos et al. [33] emphasized an increment of DMI and milk production in the cows fed using a partial mixed ration (TMR + pasture), compared to those fed using the TMR. These differences probably rely on the fact that legume silages (peas and beans, respectively) were used in TMRs’ compositions, instead of the corn silage used by us. Steinshamn [34] reported that the DMI of legume silage is higher based on the lower neutral detergent fiber (NDF) concentration of legumes and the higher rate of NDF degradation than in grasses, enabling a better balance between the metabolizable energy and the absorbed amino acids, therefore supporting a higher milk production. The TMR intake in the cows from the pTMR group based on the grazing period showed significant differences; the lowest TMR intake was recorded in the first grazing period (*p* ˂ 0.05), due to the higher pasture intake, probably caused by the lower crude fiber content of grass.

In our study, the significantly lower energy content of the pasture compared to the TMR ration (1.44 vs. 1.66 NE_L_ Mcal/kg DM, Table 1) limited milk production, also in agreement with the weight loss registered in the cows from this group (pTMR cows) compared to those fed with the TMR, which gained weight. Other studies also reported a decrease in milk yield and body weight in the grazing cows compared to those fed indoors using TMR [35,36].

Although a higher mean of daily milk yield in the TMR cows was recorded, compared to the pTMR cows (24.86 vs. 22.75 kg/d), no significant differences were registered related to fat, protein, and lactose production; these results being in line with those of other studies comparing TMR vs. pTMR [7,12,37].

Many studies proved a lower fat content of milk yielded by cows fed using TMR compared to that yielded in grazing-based feeding systems [38,39]. This statement was assigned to the high fiber content in the pasture diet, unlike the TMR diets which show a high starch content and low dietary fiber, inducing the production of high amounts of propionic acid in rumen bacteria. Such diets (high in starch and low in fiber) have been associated with fat concentration decrement in milk due to the suppression of the de novo synthesis of short- and medium-chain FAs in milk [40].

Additionally, it is well known that lactose concentration in milk is less influenced by the type of feeding [41]. In our study, a higher concentration of lactose was noticed in the milk from cows fed indoors, using the TMR. A potential explanation could reside in the high starch concentration of the TMR, resulting in a higher propionic acid throughout the rumen fermentation processes. The increment of propionate concentration in the rumen is crucial to reach higher glucose and lactose synthesis in milk [42].

Additionally, in our study, the significantly higher urea levels in the pTMR cows’ milk could be the result of a protein excess, showing a high degradability in rumen and an inadequate energy/protein ratio in pasture [11]. On the other hand, the high amounts of fermentable compounds (soluble sugars) from the pasture (especially at the beginning of the grazing—P_1_), negatively impact the normal ruminal microbial activity. Thus, nitrogen ammonia resulted from the degradation of high soluble proteins is not used and converted into microbial protein, but is mainly converted to urea nitrogen in milk and urine. A higher urea level in the milk yielded by the pTMR cows indicates a higher protein intake by using grass, but also an energy deficit [43]. O’Callaghan et al. [4] reported higher urea concentrations in the milk obtained using pastures (perennial ryegrass with white clover) rather than in that from cows fed using TMR.

Our results, concerning the influence of the grazing period on milk chemical composition, endorse those previously reported by Radonjic et al. [29] and Bohacová et al. [30], which claimed that milk’s fat content increased according to the pasture biomass maturation. The lower milk yield achieved in the P_2_ and P_3_ grazing periods is in line with the lower NE_L_ content, lower crude protein, and higher crude fiber content of grass from the pasture compared to the P_1_ period. The highest urea content in milk was reported along the P_1_ period, maybe caused by the higher protein intake, as a result of the higher legumes percentage in the pasture floristic composition, but also of the content rich in high-soluble proteins [6].

### 4.3. Fatty Acid Composition

The FA profile of milk’s fats specify its nutritional qualities. The type of feeding influenced milk fat content in OA and total MUFAs, which showed higher values in the milk yielded by the cows having access to pasture (pTMR group) compared to the TMR type of feeding. This may be the result of a higher ALA intake from grass, which is further biohydrogenated in the rumen, leading to SA formation. The SA and other FAs resulting from ALA biohydrogenation, such as VA could be unsaturated to OA and RA, respectively, in the mammary gland [43]. In our study, the higher SA and OA content of milk fat in cows having access to pasture (pTMR), and associated with their body weight loss in the experimental period, compared to the TMR cows, indicates an energy deficit in the pTMR cows’ feed and an increased mobilization of body fat reserves [7]. OA is an indicator for the intensive mobilization of body fat [44].

The OA (C18:1 *c*9) results by rumen biohydrogenation of feed’s PUFAs (mainly ALA and LA), as well as by SA desaturation (C18:0) in the mammary gland in the presence of the stearate desaturase enzyme, which is also present in the mammary tissues [45]. The fact that the Δ9-desaturase activity, in our study, does not show differences in the two groups of cows ascertains the hypothesis that the higher C18:1 *c*9 ratio in the milk fat of pTMR cows is the outcome of the mobilization of body fat reserves.

Lower concentrations of SFAs (C12:0, C14:0, and C16:0) in the milk of cows having limited access to pasture is in agreement with other authors’ statements [46,47]. This decrease is probably partly due to the higher PUFAs intake from grass, but also to the fact that the products resulted by rumen biohydrogenation of ALA from the grass are potent inhibitors of the synthesis of FAs with 10 to 14 carbon atoms in the mammary gland [48].

ALA (C18:3 n-3) content was 1.3 times higher in milk yielded by cows having access to pasture (pTMR) compared to those housed in shelter (TMR). These results are in line with the previous studies conducted by Morales-Almaraz et al. [12] and Barca et al. [7] proving a 44% and 2.64 times, respectively, higher ALA content in the milk of cows fed using pasture and TMR, compared to those fed using only TMR.

The omega-6 FA, mainly C18:2 n-6 (LA) were found in a higher concentration in the milk of cows fed using only TMR (without pastures), which was also previously reported by Morales-Almaraz et al. [12] and Barca et al. [7].

ALA (C18:3 n-3) is the upmost component in the fat of pasture, while LA (C18:2 n-6) is in the corn silage’s and concentrates’ fats [4]. Thereby, in our study, the higher ALA concentration in the milk of pTMR cows and the higher LA concentration in the milk of TMR cows, is due to the different intakes of these FAs from feed, which egressed rumen biohydrogenation.

Most of the CLA from milk is synthesized in the mammary gland based on C18:1 *t*11 throughout desaturation processes in the presence of ∆-9 desaturase [49]. The VA amount in milk was 1.48 times higher in pTMR cows. This main CLA precursor results from LA (C18:2 n-6) and linolenic acid (C18:3 n-3) incomplete rumen biohydrogenation [50], which are common in pasture fat. The lower enzymatic desaturation activity in the mammary gland of the cows having access to pasture (0.58 vs. 0.73) is consistent with the higher long-chain FA amounts in milk, resulting from body fat (mainly C18:1 *c*9) mobilization and with the higher PUFA amounts from the grass, which escaped rumen biohydrogenations and reached the duodenum, thus reducing the activity of specific enzymes in the mammary gland (∆-9 desaturation, acetyl-coA carboxylase) [7].

A higher VA and CLA *c*-9, *t*-11 content in the milk of pTMR cows suggests a healthier profile of this milk for consumers, since VA can be converted into CLA in human tissues, which shows anticancer effects [2].

The main FA group (PUFA/SFA, n-6/n-3 FA, and h/H) ratios, the AI, the TI, and the HPI are commonly used to assess milk fats quality related to the effect on consumers’ health. In general, a PUFA/SFA ratio above 0.45 and an n-6/n-3 FA ratio below 4.0 are recommended for human nutrition [51,52]. In the present study, the PUFA/SFA ratio showed considerably lower values (0.08–0.11) in both groups, than the recommended ones, while the n-6/n-3 FA ratios were within the recommended values, but only in the pTMR group of cows (1.75), based on the FA n-3 increment and the FA n-6 decrement in milk, compared to the cows fed using the TMR diet.

The consumption of milk and dairy products showing lower AI and TI values and higher h/H FA and HPI ratios, indicate benefic health effects of the cardiovascular system of consumers, but no organization has yet made recommendation in terms of values for these fat quality indices [53].

In our study, we found that cows fed on pasture (pTMR group) had significantly improved h/H FA ratio, and AI, TI, and HPI value, as a result of ALA intake increases throughout the grass, that enabled the FA n-3 content augmentation in milk. Feeding cows exclusively based on TMR revealed a negative effect on these quality indices of milk fat, due to the SFAs and LA increment and the decrement FAs considered benefic for human health.

The SFA content in milk gradually increased during the grazing period, with the highest SFA content in the third period (P_3_), mainly based on the C18:0 and C16:0 FAs’ increase. These findings support those of Radonjic et al. [29] and Bohacová et al. [30], even if Ferlay et al. [54] reported a slight decrease in the C16:0 content in milk yielded from spring to autumn, which is not consistent with our results. This could reside in pasture composition differences.

The total MUFA content decrease, including that of the individual FAs (mainly OA and VA), during the grazing period could be explained by the fact that at the same time with plants’ vegetative growing stages, C18:2 n-6 and C18:3 n-3 decreases and it is converted into C18:1 (C18:1 *c*9 and C18:1 *t*11) by rumen biohydrogenation, thereby influencing their content in milk. Similar results were reported by Radonjic et al. [29], Bohacová et al. [30], and Ferlay et al. [54].

Based on the high fiber content of grass in the third grazing period (P_3_), the milk fat content increased, next to PUFA decrease, meanwhile SFA content achieved high levels, which is an unwanted event in terms of impact on human health. Consequently, even if the milk yielded at the beginning of the grazing period shows a lower fat content, it also contains higher levels of FAs considered benefic for human health (VA, ALA, CLA). The early vegetative stage of grass showing a high ALA content (50–75%) could be the cause of the higher PUFA content in the milk produced in the first grazing period [55]. A part of the PUFAs are directly transferred into milk, whereas another part are converted into VA by rumen biohydrogenation, and further into CLA (*c*9, *t*11) in the mammary gland by desaturation [56]. Similar results of the total PUFA and individual FA content in cows’ milk, depending on the phenological vegetative phase of pasture, were disclosed also by Radonjic et al. [29] and Coppa et al. [57].

Milk fat showed a ratio of n-6/n-3 FAs, AI, and TI lower values at the beginning of grazing, whereas the most critical values of sanogenous lipid indices were registered at the end of the grazing period, maybe as a result of the chemical composition change of grass and probably of the energy deficit recorded as a consequence of decreased intake and digestibility of grass nutrients.

### 4.4. Fat-Soluble Vitamins and Antioxidant Capacity

The results of this study reveal significantly higher α-tocopherol, β-carotene, and retinol concentrations in the milk of cows that grazed and received TMR-supplemented feed in the shelter; similar results were also reported by other researchers [33,58]. A four times higher concentration of fat-soluble vitamins and β-carotene in the milk of cows fed on pasture, compared to those fed indoors using TMR, was reported by Butler et al. [59]. In our study, the increase in fat-soluble antioxidant concentrations in the grazing cows’ milk (pTMR group) had lower values (2–2.5 times), since the grazing time was limited to 8 h/day.

The highest milk antioxidant activity (μM TE/mL) was associated with the highest concentration of α-tocopherol (0.74 mg/100 g), retinol (125.62 μg/100 g), and β-carotene (0.69 μg/100 g) determined in the cows’ milk of the pTMR group, which had access to pasture. These results backup the conclusions of Stobiecka et al. [60] claiming that the increase in the fat-soluble antioxidants content showed the same trend as milk antioxidant activity, which increased too.

Additionally, in our study, the α-tocopherol and retinol concentrations insignificant diminution in the pasteurized milk could be explained by the low temperature used at pasteurization (65 °C) [15]. The lowering of natural antioxidants concentration in the milk stored for four days in the refrigerator could be justified by the fact that this period accounts for the formation of reactive oxygen species, which are inactivated by milk antioxidants. By means of this function, α-tocopherol, retinol, and β-carotene are oxidized, thus conducting to a decrease in their concentration in milk [61].

TAC (µmol TE/mL) showed lower values in heat-treated milk rather than in raw milk, significant differences being observed only in the case of the pTMR group. Calligaris et al. [62] stated that the pasteurization process (at 90 °C for 10 min.) led to an increased pro-oxidant activity, probably because of the breakup of some natural antioxidants (tocopherols, vitamin A, carotenes) and the formation of new oxidative molecules during Maillard reactions. Furthermore, Yilmaz-Ersan et al. [63] noted that heat treatment conditions (temperature and time) and milk composition in natural antioxidants influence milk antioxidant capacity, which could endorse the results of our study, in view of a lower lipophilic antioxidants content in TMR milk. These results suggest that the milk obtained from pTMR cows had higher nutritional qualities than the one yielded by cows fed indoors, using TMR.

The antioxidant activity does not change significantly during storage, although it tends to decrease slightly. Accordingly, consumers may enjoy the benefits of milk’s nutritional qualities and bioactive compounds during the four days of storage of the pasteurized milk in the refrigerator.

The mean values of milk samples TAC in the present study were higher than those reported by other authors [64,65], probably as outcome of the higher fat concentration of the Jersey cows’ milk, used in our study. Moreover, it is well known that milk TAC is influenced by heat treatment, but also by fat concentration [66].

## 5. Conclusions

The use of pastures use in cows’ diets, combined with the TMR, provided *ad libitum* indoors, could serve as a strategy to improve milk FA profiles, lipophilic antioxidant content and TAC. Eight hours of grazing per day combined with a ration supplementation using a TMR provided ad libitum indoors, could produce a milk with a higher content of FAs benefic for human health (VA, CLA, and n-3 FA), and lipophilic antioxidants (α-tocopherol, retinol, and β-carotene). Such a feeding regimen could also lead to saving up important TMR quantities (about 4.3 kg DM/day/cow).

The pTMR (partial mixed ration: grazing + TMR) feeding system relies on a compromise between the quantitative production and the quality of the milk, while providing an enhanced valorization of the available pastures. The FA profile (VA, CLA, and ALA concentration) and the fat-soluble antioxidants content may be a mark of cows feeding on pasture, having a positive effect on the consumers’ perception and acceptance of milk and dairy products. Additionally, under the conditions of our experiment, cows fed indoors using a TMR based on corn silage and concentrates showed higher milk yields, but lower bioactive compound concentrations in milk.

## Figures and Tables

**Table 1:** Chemical composition, fatty acid profile and fat-soluble antioxidants content of TMR and pasture in the different trial periods.

Item		TMR	Pasture	Grazing Period ^#^			SEM	*p*-Values	
				P_1_	P_2_	P_3_		S	P
Chemical composition (% of DM):									
DM (dry matter), %		53.65	31.53	27.15 ^c^	32.90 ^b^	34.55 ^a^	0.217	***	**
Crude protein (CP)		17.26	14.78	15.74 ^a^	14.40 ^b^	13.71 ^b^	0.181	**	*
OM (organic matter)		91.77	92.07	91.70	92.12	92.40	0.157	n.s.	n.s.
Ether extract (EE)		2.69	2.89	2.82	2.93	2.92	0.031	n.s.	n.s.
Crude fiber (CF)		15.85	28.10	26.71 ^b^	28.17 ^a^	29.92 ^a^	0.176	***	**
Nitrogen free extractive (NFE) ^1^		55.97	46.30	46.43	46.62	45.85	0.417	**	n.s.
Ca ^2^		0.76	-	-	-	-	-		
P ^2^		0.37	-	-	-	-	-		
NE_L_ (Mcal/kg DM) ^2^		1.66	1.44	1.47	1.44	1.42	0.005	**	n.s.
Fatty acids profile (% of total FA):									
Total FA (g/kg DM)		15.84	17.81	21.74 ^a^	17.48 ^b^	14.22 ^c^	0.236	*	**
	C16:0	14.91	13.88	12.80 ^b^	13.75 ^b^	15.10 ^a^	0.417	n.s.	*
	C18:0	7.32	2.20	1.78	2.06	2.75	0.194	***	n.s.
	C18:1 *cis*-9	18.22	1.71	1.38	1.78	1.97	0.210	***	n.s.
	C18:2 *cis*-9, *cis*-12	39.82	12.18	10.42 ^c^	12.02 ^b^	14.10 ^a^	0.308	***	*
	C18:3 *cis*-9, *cis*-12, *cis*-15	8.65	64.47	68.10 ^a^	64.77 ^b^	60.54 ^c^	1.172	***	***
	C18:2/C18:3	4.60	0.19	0.15 ^b^	0.19 ^ab^	0.23 ^a^	0.000	***	**
	Other	11.08	5.56	5.52	5.62	5.54	0.343	***	n.s.
Fat-soluble antioxidants (mg/kg DM):									
	α-tocopherol	8.64	26.91	34.41 ^a^	26.14 ^b^	20.18 ^c^	0.447	***	***
	All-*trans* β-carotene	6.73	66.97	82.32 ^a^	67.12 ^b^	51.47 ^c^	0.851	***	***

TMR: total mixed ration; TMR composition (% of DM): Corn silage—42.0; Alfalfa hay—10.0; Grass hay—7.3; Corn grain—10.0; Triticale—5.0; Soybean meal—9.0; Rape-seed meal—12.0; Sodium bicarbonate—0.3; Salt—0.4; Vitamin and mineral premix—4.0. SEM: standard error of the mean. S—TMR vs. pasture, P—effect of the grazing period. ^1^ Calculated by difference 100—(%CF + %CP + %EE + %ash). ^2^ Estimated using values of NRC [17]. ^#^ Grazing period (3 weeks): P_1_—May, P_2_—June, P_3_—June/July. ^a–c^ Mean values within a row without a common superscript differ significantly (*p* < 0.05). Statistically significant difference: * *p* < 0.05; ** *p* < 0.01; *** *p* < 0.001; n.s.—not significant.

**Table 2:** Effect of cow feeding system (S: TMR vs. pTMR) and grazing period (P) on body weight (BW) and feed intake.

Item		TMR	pTMR	Grazing Period ^#^			SEM	*p*-Values	
				P_1_	P_2_	P_3_		S	P
Body weight (kg):									
	- initial	526	520	520	516	514	24.3	n.s.	n.s.
	- final	535	514	516	514	514	27.1	**	n.s.
	- change	+9	−7	−4 ^a^	−2 ^ab^	−1 ^b^	0.56	***	*
Energy requirements—NE_L_ (Mcal/day) ^1^:									
	- maintenance	8.60	8.60	8.60	8.60	8.60	-	-	-
	- milk production	20.63	19.79	20.64	19.70	18.72	-	-	-
	- movement	-	2.0	2.0	2.0	2.0	-	-	-
	- change of BW	+0.83	−0.46	−0.97	−0.49	−0.25	-	-	-
Dry Matter Intake (kg/day)									
	- TMR, from which:	18.11	13.80	13.05 ^b^	13.50 ^a^	13.74 ^a^	0.418	***	*
	- forage	10.73	8.18	7.74	8.00	8.15	0.283	**	n.s.
	- concentrate	7.38	5.62	5.31	5.50	5.59	0.382	**	n.s.
	- pasture	-	4.82	5.85 ^a^	5.07 ^b^	4.46 ^c^	0.291	-	**
	Total: —kg DM/day	18.11	18.62	18.90	18.57	18.20	0.415	n.s.	n.s.
	—kg DM/100 kg BW	3.42	3.60	3.65	3.60	3.54	0.132	n.s.	n.s.
NE_L_ (Mcal/day)									
	- Total, from which:	30.06	29.93	30.27	29.81	29.32	1.072	n.s.	n.s.
	- TMR	30.06	22.90	21.67	22.41	22.81	0.985	***	n.s.
	- pasture	-	7.03	8.60 ^a^	7.40 ^b^	6.51 ^c^	0.268	-	**
	Differences from the requirements	+0.83	−0.46	−0.97	−0.49	−0.25	-	-	-
Feed conversion ratios (FCR)									
	NE_L_ (Mcal/kg milk), from which:	1.21	1.32	1.28	1.32	1.36	0.059	*	n.s.
	- TMR	1.21	1.01	0.92	0.99	1.06	0.055	*	n.s.
	- pasture	-	0.31	0.36	0.33	0.30	0.006	-	*
	Concentrate (g/kg milk)	296.8	247.0	223.8	242.9	259.7	8.421	**	*

TMR: total mixed ration; pTMR: partial mixed ration; SEM: standard error of the mean; ^1^ Estimated according to NRC values [17]. # Grazing period (3 weeks): P_1_—May, P_2_—June, P_3_—June/July. ^a–c^ Mean values within a row without a common superscript differ significantly (*p* < 0.05). Statistically significant difference: * *p* < 0.05; ** *p* < 0.01; *** *p* < 0.001; n.s.—not significant.

**Table 3:** Effect of cow feeding system (S: TMR vs. pTMR) and grazing period (P) on milk production and composition.

Item		TMR	pTMR	Grazing Period ^#^			SEM	*p*-Values	
				P_1_	P_2_	P_3_		S	P
Raw milk yield (kg/d)		24.86	22.75	23.72 ^a^	22.64 ^b^	21.52 ^c^	0.555	*	**
Corrected milk yield:									
	- FCM—3.5% (kg/d)	28.81	28.14	28.64	27.67	27.49	0.622	n.s.	n.s.
	- ECM (kg/d)	29.23	28.36	29.21 ^a^	27.75 ^ab^	27.33 ^b^	0.528	n.s.	*
Milk content (%):									
	- fat	4.48	4.96	4.78 ^b^	4.87 ^b^	5.21 ^a^	0.202	**	*
	- protein	3.91	4.04	4.15	3.90	3.91	0.094	n.s.	n.s.
	- lactose	4.72	4.58	4.44	4.53	4.65	0.045	*	n.s.
	- urea-N	19.80	25.21	28.72 ^a^	25.80 ^b^	24.62 ^b^	0.850	**	*
	- SNF	9.46	9.69	9.57	9.23	9.45	0.097	n.s.	n.s.
Milk yield (kg/d):									
	- fat	1.114	1.128	1.133	1.103	1.121	0.038	n.s.	n.s.
	- protein	0.972	0.919	0.984 ^a^	0.883 ^b^	0.841 ^b^	0.023	n.s.	*
	- lactose	1.173	1.042	1.053	1.026	1.001	0.028	n.s.	n.s.
SCC (×1000 cells/mL)		143.7	245.6	285.3	254.4	195.2	21.43	**	*

TMR: total mixed ration; pTMR: partial mixed ration; SEM: standard error of the mean; SNF: solids non-fat; SCC: somatic cells count; FCM: fat-corrected milk and ECM: energy-corrected milk—calculated according to NRC equations [17]. ^#^ Grazing period (3 weeks): P_1_—May, P_2_—June, P_3_—June/July. ^a–c^ Mean values within a row without a common superscript differ significantly (*p* < 0.05). Statistically significant difference: * *p* < 0.05; ** *p* < 0.01; n.s.—not significant.

**Table 4:** Effect of cow feeding system (S: TMR vs. pTMR) and grazing period (P) on milk fatty acid (FA) composition.

FA, %		TMR	pTMR	Grazing Period ^#^			SEM	*p*-Values	
				P_1_	P_2_	P_3_		S	P
C4:0		1.20	1.15	1.39	1.30	1.09	0.114	n.s.	n.s.
C6:0		1.51	1.49	1.54 ^a^	1.42 ^ab^	1.21 ^b^	0.110	n.s.	*
C8:0		0.98	0.99	1.20	1.15	1.15	0.102	n.s.	n.s.
C10:0		2.54	2.29	2.73	2.77	2.41	0.314	n.s.	n.s.
C12:0		2.76	2.47	4.01	3.36	3.22	0.261	n.s.	n.s.
C14:0		11.37	9.51	8.26	9.92	9.56	0.512	*	n.s.
C14:1		1.07	1.22	0.98	1.15	1.18	0.034	n.s.	n.s.
C16:0		31.24	28.41	25.69 ^b^	27.99 ^a^	28.82 ^a^	1.437	*	**
C16:1		1.38	1.54	1.57	1.56	1.53	0.122	n.s.	n.s.
C18:0		12.75	13.61	13.33 ^b^	13.71 ^ab^	14.69 ^a^	0.726	*	*
C18:1 *trans*-11 (VA)		1.40	3.46	4.00 ^a^	3.20 ^b^	2.59 ^c^	0.389	***	**
C18:1 *cis*-9 (OA)		23.01	24.23	24.71 ^a^	22.69 ^b^	22.90 ^b^	0.507	*	*
C18:2 *cis*-9, *cis*-12 (LA)		3.36	2.73	2.90 ^b^	2.86 ^b^	3.44 ^a^	0.052	*	*
Total CLA		1.07	2.10	2.58 ^a^	1.83 ^b^	1.96 ^b^	0.283	**	*
*cis*-9, *trans*-11 CLA (RA)		0.99	2.01	2.49 ^a^	1.75 ^b^	1.84 ^b^	0.008	***	**
*trans*-10, *cis*-12 CLA		0.07	0.08	0.09	0.08	0.08	0.004	n.s.	n.s.
C18:3 *c*9, *c*12, *c*15 (ALA)		0.58	1.33	1.54 ^a^	1.30 ^b^	1.27	0.091	***	*
C20:4 (AA)		0.16	0.13	0.14	0.10	0.11	0.004	n.s.	n.s.
C20:5 n-3 (EPA)		0.08	0.14	0.14	0.12	0.11	0.006	*	n.s.
C22:5 n-3 (DPA)		0.12	0.19	0.20	0.18	0.18	0.008	*	n.s.
Unidentified fatty acids		3.22	2.93	3.09	3.39	3.08	0.054	n.s.	n.s.
SFA		64.54	59.95	58.65 ^b^	61.62 ^a^	62.15 ^a^	0.610	**	**
UFA		32.23	37.07	38.76 ^a^	35.49 ^b^	35.77 ^b^	0.851	**	*
MUFA		26.88	30.46	31.26 ^a^	29.10 ^b^	29.20 ^b^	0.376	**	*
PUFA		5.35	6.61	7.50 ^a^	6.39 ^b^	6.57 ^b^	0.217	**	*
hFA		29.81	34.33	36.21 ^a^	32.28 ^b^	32.56 ^b^	0.725	***	**
HFA		45.39	40.39	37.96 ^b^	41.27 ^a^	41.60 ^a^	0.854	**	**
n-3 FA		0.79	1.67	1.88 ^a^	1.60 ^b^	1.56 ^b^	0.032	**	*
n-6 FA		3.52	2.86	3.04	2.96	3.55	0.112	*	n.s.
Product/substrate ratios:									
	C14:1/C14:0	0.097	0.13	0.119	0.116	0.123	0.008	n.s.	n.s.
	C16:1/C16:0	0.044	0.055	0.061	0.056	0.053	0.003	n.s.	n.s.
	*c*-9 C18:1/C18:0	1.83	1.82	1.85 ^a^	1.65 ^ab^	1.56 ^b^	0.193	n.s.	*
	*c*-9, *t*-11 CLA/*t*-11 C18:1	0.73	0.58	0.62 ^b^	0.55 ^b^	0.71 ^a^	0.028	*	*
	∆^9^-desaturase index	0.318	0.346	0.37	0.33	0.33	0.043	n.s.	n.s.

TMR: total mixed ration; pTMR: partial mixed ration; ^#^ Grazing period (3 weeks): P_1_—May, P_2_—June, P_3_—June/July; SEM: standard error of the mean; VA: vaccenic acid; OA: oleic acid; LA: linoleic acid; CLA: conjugated linoleic acid; RA: rumenic acid; ALA: α-linolenic acid; AA: arachidonic acid; EPA: eicosapentaenoic acid; DPA: docosapentaenoic acid; hFA: hypocholesterolemic FA; HFA: Hypercholesterolemic FA. ^a–c^ Mean values within a row without a common superscript differ significantly (*p* < 0.05). Statistically significant difference: * *p* < 0.05; ** *p* < 0.01; *** *p* < 0.001; n.s.—not significant.

**Table 5:** Effect of cow feeding system (S: TMR vs. pTMR) and grazing period (P) on health lipid indices in milk.

Parameter	TMR	pTMR	Grazing Period ^#^			SEM	*p*-Values	
			P_1_	P_2_	P_3_		S	P
PUFA/SFA	0.082	0.110	0.128 ^a^	0.104 ^b^	0.106 ^b^	0.008	*	*
n-6/n-3 FA	4.45	1.75	1.62 ^b^	1.85 ^b^	2.27 ^a^	0.398	***	**
AI	2.55	1.97	1.73 ^b^	2.11 ^a^	2.05 ^a^	0.114	*	*
TI	3.49	2.79	2.48 ^b^	2.92 ^a^	3.00 ^a^	0.095	**	*
h/H	0.65	0.85	0.95 ^a^	0.78 ^b^	0.78 ^b^	0.032	*	*
HPI	0.68	0.87	0.95 ^a^	0.82 ^b^	0.82 ^b^	0.026	*	*

TMR: total mixed ration; pTMR: partial mixed ration; ^#^ Grazing period (3 weeks): P1—May, P2—June, P3—June/July; SEM: standard error of the mean; AI: Atherogenicity Index; TI: Thrombogenicity Index; h/H (hypocholesterolemic/Hypercholesterolemic ratio); HPI: Health Promoting Index. ^a,b^ Mean values within a row without a common superscript differ significantly (*p* < 0.05). Statistically significant difference: * *p* < 0.05; ** *p* < 0.01; *** *p* < 0.001.

**Table 6:** Effect of cow feeding system (S: TMR vs. pTMR) on antioxidant content and TAC of raw, pasteurized and stored milk.

Parameter	Groups	Milk		
		Raw	Pasteurized	Stored 4 Days
α-tocopherol (mg/100 g)	TMR	0.27 ± 0.02 ^y^	0.24 ± 0.03 ^y^	0.24 ± 0.02 ^y^
	pTMR	0.74 ± 0.03 ^a,x^	0.68 ± 0.03 ^a,x^	0.63 ± 0.02 ^b,x^
Retinol (µg/100 g)	TMR	57.51 ^a^ ± 1.39 ^y^	50.82 ± 1.15 ^ab,y^	49.74 ± 1.35 ^b,y^
	pTMR	125.62 ± 3.81 ^a,x^	101.31 ± 2.95 ^a,x^	97.82 ± 1.64 ^b,x^
All-*trans* β-carotene (µg/100 g)	TMR	0.41 ± 0.02 ^y^	0.37 ± 0.04 ^y^	0.35 ± 0.02 ^y^
	pTMR	0.69 ± 0.04 ^a,x^	0.55 ± 0.03 ^b,x^	0.42 ± 0.04 ^c,x^
TAC (µmol TE/mL)	TMR	2.53 ± 0.21 ^y^	2.47 ± 0.27 ^y^	2.40 ± 0.18
	pTMR	3.02 ± 0.43 ^a,x^	2.73 ± 0.24 ^b,x^	2.58 ± 0.35 ^b^

TMR: total mixed ration; pTMR: partial mixed ration. TAC = Total Antioxidant Capacity; TE = Trolox Equivalent; ^a–c^ Mean values within a row without a common superscript differ significantly (*p* < 0.05); ^x,y^ Mean values within a column without a common superscript corresponding to a parameter differ significantly (*p* < 0.05).

## Data Availability

Data are available upon request by contacting the corresponding author.
